# Comparative study of the fungicide Benomyl toxicity on some plant growth promoting bacteria and some fungi in pure cultures

**DOI:** 10.2478/intox-2014-0002

**Published:** 2014-07-16

**Authors:** Randa H. Elslahi, Awad G. Osman, Ashraf M. Sherif, Adil A. Elhussein

**Affiliations:** 1 Biofertilization Department, Environment and Natural Resource Research Institute, National Center for Research, Khartoum, Sudan; 2 Botany Department, Faculty of Science, University of Khartoum, Khartoum, Sudan

**Keywords:** *Aspergillus niger*, *Penicillium* sp., *Fusarium oxysporum*, Benomyl, toxicity

## Abstract

Six laboratory experiments were carried out to investigate the effect of the fungicide Benomyl on pure cultures of some plant growth promoting bacteria (PGPB) and some fungi. The highest LD_50_ was recorded for *Bacillus circulans* and proved to be the most resistant to the fungicide, followed by *Azospirillum braziliense*, while *Penicillium sp.* was the most affected microorganism. LD_50_ values for the affected microorganisms were in 21–240 orders of magnitude lower in comparison with the LD_50_ value for *Azospirillum braziliense*. The results indicate a strong selectivity for Benomyl against *Rhizobium meliloti* and *Penicillium sp.* when compared to other microorganisms tested. The highest safety coefficient was recorded for *Bacillus circulans* followed by *Azospirillum braziliense*, while *Rhizobium meliloti*, showed the lowest safety coefficient value compared to other bacteria. The lowest toxicity index was recorded for *Bacillus circulans* and *Azospirillum braziliense*. The slope of the curves for *Bacillus sp.* and *Rhizobium meliloti* was steeper than that of the other curves, suggesting that even a slight increase of the dose of the fungicide can cause a very strong negative effect. In conclusion, Benomyl could be applied without restriction when using inocula based on growth promoting bacteria such as symbiotic nitrogen fixers (*Rhizobium meliloti*), non-symbiotic nitrogen fixers (*Azospirillum braziliense*) or potassium solibilizers (*Bacillus circulans*), given that the fungicide is applied within the range of the recommended field dose.

## Introduction

Due to continuous use of pesticides, appreciable quantities of them and their degradation products may accumulate in the ecosystem. Prevailing data showed that only 2–3% of the applied chemical pesticides reach their targets, while the rest remains in the soil (US-EPA, 2005). Their excessive use causes serious damage to the ecosystem, terrestrial as well as aquatic, and consequently to the flora and fauna of the surroundings (Paliwal *et al.,* 2009). This raises great alarm about the heavy contamination burden the soil is receiving. A great risk is being posed on soil microbes and there is interference with element cycles and entry into food chains. Among the pesticides used in Sudan, fungicides rank third after insecticides and herbicides. Fungicides were found to have the largest inhibition effect on soil microorganisms (Kruglov, 1991). One of the recently introduced fungicides in Sudan is Benlate, which is the commercial name for the active ingredient Benomyl or Methyl 1-(butylcarbamoyl)benzimidazole-2-ylcarbamate. It belongs to the benzimidazole family, a member of the carbamate group. It is selectively toxic to microorganisms and invertebrates. It is a systemic broad spectrum, protective and eradicant fungicide used for the control of many plant fungal pathogens and cold storage rots. The controlled fungi are mainly those causing powdery mildews, *Botrytis, Fusarium* basal rot, black spot and blossom rot. In Sudan it is used for the treatment of powdery mildews mainly in cucurbits and other vegetables. Seed protection and seed inoculation are frequently incompatible. One way of allowing for the successful infection of legume roots with *Rhizobium* after treatment of seeds with fungicides is to use a fungicide-resistant inoculant (Odeyemi & Alexander, 1977). The objective of this study is to investigate the toxicity of the fungicide Benomyl on some plant growth promoting bacteria and some fungi in pure cultures.

## Materials and methods

### The effect of Benomyl on pure cultures of nitrogen fixing bacteria

(*Rhizobium meliloti, Azospirillum braziliense),* potassium solubilizing bacteria *(Bacillus circulans),* and some fungi (*Aspergillus niger, Penicillium* sp . and *Fusarium oxysporum*) was evaluated by determining LD_50_, Benomyl effective concentration limits, Benomyl selectivity index (SI), Benomyl safety coefficient (SC) and toxicity index (TI). Benomyl (50% wettable powder) (M.wt: 290.3).

All microorganisms used were obtained from the Environment and Natural Resources Research Institute (ENRRI), National Centre for Research (NCR) – Khartoum – Sudan.

Two different media, Meat Peptone Agar and Czapeks Dox Agar were prepared by dissolving the ingredients of each (g) in one liter of distilled water as follows: **Meat Peptone Agar (MPA**)**:** Meat extract 5.0; Peptone 7.5; Sodium chloride 5.0 and Agar 20.0. **Czapeks Dox Agar (CZA):** Sucrose 20.0; Sodium 2.0; Dipotassium hydrogen phosphate 1.0; Magnesium sulphate, hydrated (MgSO_4_.7H_2_O) 0.5; Potassium chloride 0.5; Calcium carbonate 3.0 and Agar 20.0 (Tepper *et al.,* 1993).

Benomyl effective concentration limits (20–80%) for *Azospirillum braziliense*, *Rhizobium meliloti* and *Bacillus circulans* were determined as suggested by Zinchenko *et al.* (1974). Each bacterial strain was grown on meat peptone broth for 24 hours, the culture was serially diluted and 0.5 ml of the proper dilution chosen for each microbe, was transferred to inoculate plates of MPA supplemented with different Benomyl concentrations. The plates were incubated at 28°C for 48 hours and then the observed colonies were counted. Benomyl concentrations used (g/L), suggested for each microbe according to its effective concentration limits, were: 3.83, 4.83, 5.33 and 6.33 for *Rhizobium* *meliloti*, 0.033, 0.233, 1.332 and 2.331 for *Azospirillum braziliense,* and 2.3, 2.8, 3.3, 3.8 and 4.8 for *Bacillus circulans*. A control set of MPA plates which were not supplemented with Benomyl was prepared for comparison. The concentrations of the fungicide that caused 50% destruction (LD_50_) of the cells of pure cultures of the microorganisms were calculated by log-dose/probit regression line method Finney (1971) using computer software (Biostat, 2008).

For determining Benomyl effective concentration limits for fungi, *Fusarium oxysporum*, *Aspergillus niger* and *Penicillium* sp . were grown onto Czapeks Dox Agar plates for ten days and 1.1cm discs were then cut and seeded onto the surface of CZA plates (Shattock, 1988), which were previously supplemented with different Benomyl concentrations. Benomyl concentrations used were: 0.003, 0.0033, 0.0066, 0.01, 0.013 and 0.017 for both of *Aspergillus niger* and *Penicillium* sp ., and 0.003, 0.01, 0.02, 0, 03 and 0.04 for *Fusarium oxysporum*. Control sets were included for comparison. Ten days later, the growth diameters in the treated and control plates were measured and recorded in cm.

Calculated LD_50_ for each bacterial and fungal strain was used to calculate Benomyl selectivity Index (SI) and safety coefficient (SC) following Kruglov (1991):

SI=(LD_50_ of the first Microorganism)/(LD_50_ of the second Microorganism)

SC=(LD_50_)/(Field dose)

Toxicity index (TI) of Benomyl was determined according to Sun (1950).

## Results

**Fig. 1:**
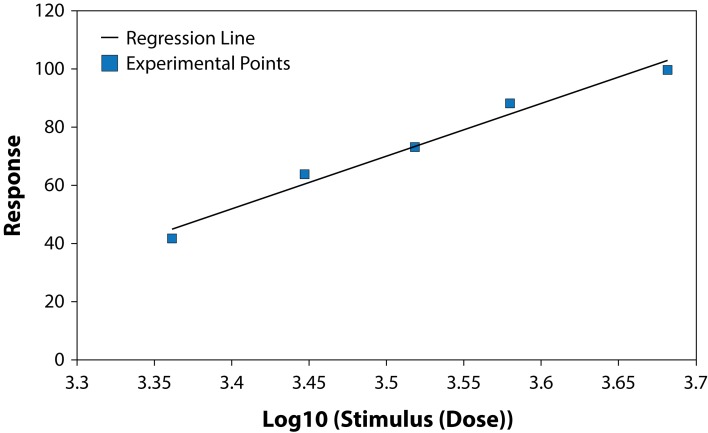
Dose-effect relationship for *Bacillus circulans.*

**Fig. 2:**
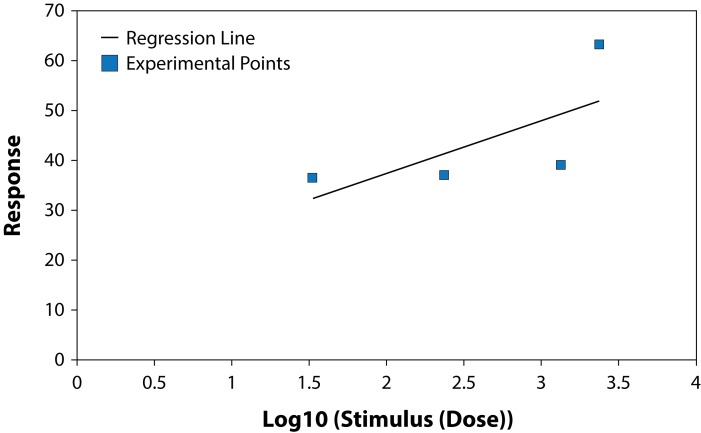
Dose-effect relationship for *Azospirillum* sp.

**Fig. 3:**
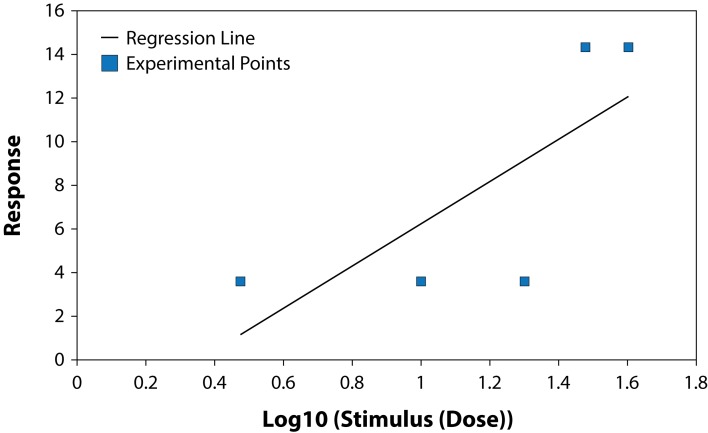
Dose-effect relationship for *Fusarium oxysporum* sp.

**Fig. 4:**
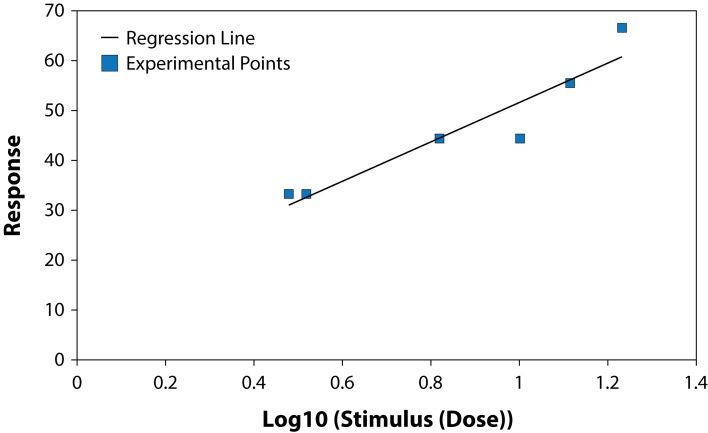
Dose-effect relationship for *Aspergillus niger.*

**Fig. 5:**
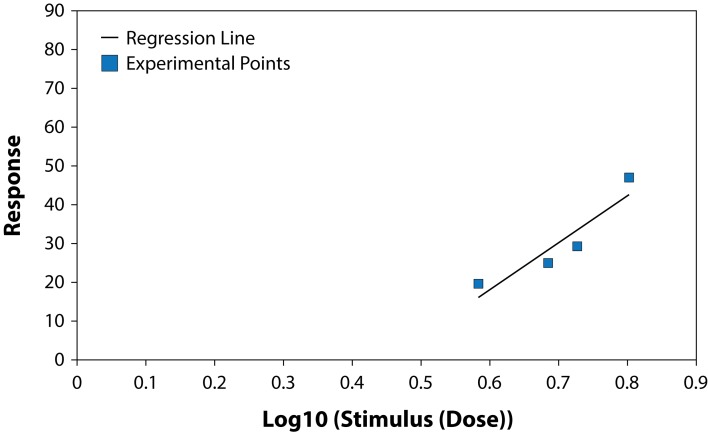
Dose-effect relationship for *Rhizobium* sp.

**Fig. 6:**
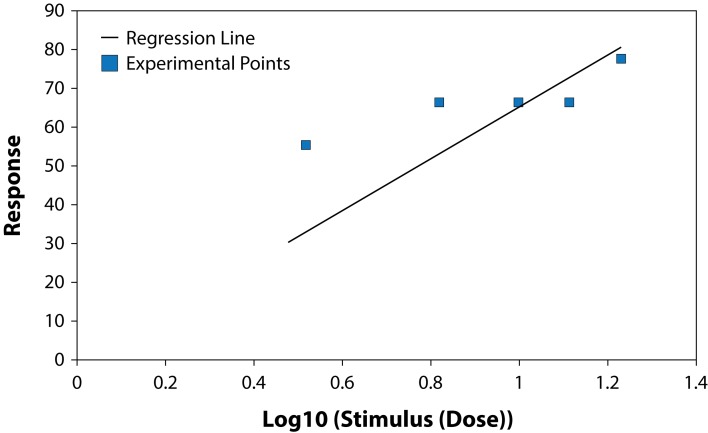
Dose-effect relationship for *Penicillium* sp.

The results of studying the influence of the fungicide Benomyl on growth and development of pure cultures of PGPB and some fungi are presented in Tables 1 and 2, (Figures 1–6). The highest LD_50_ (2528.74 ppm) was recorded for *B. circulans* and the lowest (6.01 ppm) for *Penicillium* sp. (Tables 1 and 2). LD_50_ values for the affected microorganisms were in 21–240 orders of magnitude lower in comparison with LD_50_ value for *Azospirillum braziliense. Rhizobium meliloti, Azospirillum braziliense, Bacillus circulans, Aspergillus niger, Penicillium* sp. and *Fusarium oxysporum* showed different resistance to Benomyl with selectivity indexes (SI) in the range of 1.71–420.76. (Table 1). These results indicate that Benomyl has a strong selectivity against *R. meliloti* and *Penicillium* sp. when compared to the other microorganisms tested. The safety coefficient of the most sensitive microorganism to Benomyl is more than 2000 compared to the other microorganisms tested. The highest safety coefficient (842913) was recorded for the potassium solubilizing bacterium *B. circulans,* followed by the free nitrogen fixing bacteria *A. braziliense*, while the symbiotic nitrogen fixer R. *meliloti* showed a low safety coefficient value of 2346 compared to other microorganisms, except *Penicillium* sp. The lowest toxicity index was recorded for *B. circulans* and *Azospirillum braziliense* while the highest was recorded for *Penicillium* sp. and *Rhizobium meliloti.* The slope of the curves for *Bacillus* sp. and *Rhizobium meliloti* is steeper than that of the other curves, suggesting that even a slight increase in the fungicide dose can cause a very strong negative effect on the growth of these microorganisms (Figures1–6).

**Table 1:** Effect of Benomyl on pure cultures of different micro-organisms.

		Index of Selectivity					
		1	2	3	4	5	6
Species	LD_50_ (ppm)	*B. circu*	*Azospir*	*Fusar*	*Aspergill*	*Rhizob*	*Penicell*
*B. circulans*	2528.74		1.75	37.27	280.04	359.20	420.76
*Azospirillum*	1444.87			21.30	160.01	205.24	240.41
*Fusarium*	67.85				7.51	9.64	11.29
*Aspergillus*	9.03					1.28	1.50
*Rhizobium*	7.04						1.17
*Penicellium*	6.01						

**Table 2:** Inhibition effect of Benomyl on growth of different micro-organisms.

No	Microorganisms	LD_50_ (ppm)	Safety Coefficient	Toxicity Index (%)
1	*B. circulans*	2528.74	842913	0.24
2	*Azospirillum*	1444.87	481623	0.42
3	*Fusarium*	67.85	22616	8.86
4	*Aspergillus*	9.03	3010	66.56
5	*Rhizobium*	7.04	2346	85.37
6	*Penicillium*	6.01	2003	100

## Discussion

The most sensitive microorganisms towards Benomyl, as determined by LD_50_, were *Rhizobium meliloti* and *Penicillium* sp, as compared to the other microorganisms tested. The most tolerant was *Bacillus circulans,* followed by the free nitrogen fixing bacterium *Azospirillum braziliense.* Osman *et al.* (2012) found the most sensitive microorganisms towards thiram, as determined by LD_50_, to be *Azospirillum* and *Pseudomonas aurentiaca, w*hile the most tolerant were *Falvobacterium* followed by *Fusarium oxysporum*, *Azomonas* and *Rhizobium meliloti*. LD_50_ values for the affected microorganisms were in 21–240 orders of magnitude lower in comparison with the LD_50_ value for *Azospirillum braziliense.* Kalinin *et al.* (2002) found that EC_50_ values for resistant microorganisms towards azoxystrobin were in 3–5 orders of magnitude higher in comparison with EC_50_ values for sensitive strains.

Srinivasulu *et al.* (2012 reported that Monocrotophos and Chlorpyrifos pesticides caused a stimulatory effect on *Azospirillum* sp. at doses of 2.5 to 5 kg/ha in laterite and vertisol soils. Diuron and Chlorotoluron were found to cause no effect on nitrogen fixers while Linuron caused a strong effect. Glyphosate and Methamidophos were found to stimulate soil microbial growth, whereas Fenamephos was detrimental to nitrification bacteria (Lo, 2010).

When Carbofuran, Chlormephos, Terbufos and Benfuracarb were tested for compatibility with *Azospirillum lipoferum* on solid cultures, only Terbufos was found to induce a slight effect on growth (Revellin *et al.*, 2001). Gomez *et al.* (1998) found that the pesticide Promopropylate did not affect the cell growth of *Azospirillum braziliense,* while it was significantly reduced by Methidathion in chemically defined media at 10, 50, 100, 200 and 300µg/ml.

Brominal, Cuprisal and Fenvalerate pesticides were found to suppress growth of *Azospirillum chroococcum, Azospirillum braziliense* and *Azospirillum lipoferum* at 10 and 50 ppm (Omar & Abd-Alla, 1992).

Results of this study showed different resistance of the microorganisms tested to Benomyl, with selectivity indexes (SI) in the range of 1.71–420.76. Osman *et al.* (2012) found selectivity indexes ranging from 1.496 to 7447.5 for the fungicide Thiram against different microorganisms. The safety coefficient of the most sensitive microorganism to Benomyl is more than 2000. According to Kalinin *et al.* (2002), a fungicide is considered safe for a given microorganism when its safety coefficient is more than 15. The safety coefficients for the microorganisms tested were within the range of 2003 to 842913. This indicates that the fungi tested might have developed resistance against Benomyl. Similar observations and conclusions were also drawn by (Cooksey, 1990 and Ogawa *et al.,* 1983) for a number of fungi, including *Aspergillus nidulans, Erysiphe* spp. *Penicillium* spp. and *Fusarium* spp., against Benomyl. Fravel *et al.* (2005) found that the fungicide Thiram at concentrations of 10, 30, 50 or 100ppm a.i. did not kill *Fusarium oxysporum* strain CS-20 in the *in vitro* experiment, but it was most toxic to the fungus and significantly reduced its growth rate and final colony size at 30 ppm or greater. The lowest toxicity index was recorded for *B. circulans* and *Azospirillum braziliense,* while the highest toxicity indices were recorded for *Penicillium* sp and *Rhizobium meliloti.* Daoud *et al.* (1990) found that the fungicide Benomyl was the most toxic of the pesticides tested against *Alternaria* spp. followed by fluazifop and Decis (deltamethrin). Osman *et al.* (2012) found that thiram was most toxic to *Pseudomonas aurentiaca* followed by *Azospirillum*. The lowest toxicity index was recorded for *Fusarium oxysporum* and *Flavobacterium*. The slope of the curves for *Bacillus* sp. and *Rhizobium meliloti* is steeper compared to the other curves, suggesting that even a slight increase of the dose of the fungicide can cause a very strong negative effect. Kalinin *et al.* (2002) found that the slope of the dose-reaction curve for *Klebsiella planticola* was steeper than that of the curves for *Pseudomonas putida*, *Azotobacter chrococcum* and *Clostridium acetobutilicum*.

The results presented here indicate that Benomyl can be used in association with the microbial inoculants of biological nitrogen fixers and potassium solubilizers.
